# FIB-4 Index as a Non-Invasive Biomarker of Congestion and Prognosis in Heart Failure

**DOI:** 10.3390/jcm15124448

**Published:** 2026-06-09

**Authors:** Amato Serra, Emilio Palmieri, Maria Livia Burzo, Mariella Fuorlo, Antonio De Vita, Marcello Covino, Giovanni Gambassi, Giuseppe De Matteis

**Affiliations:** 1 Department of Internal Medicine, Fondazione Policlinico Universitario A. Gemelli IRCCS, 00168 Rome, Italy; amatoserra@gmail.com (A.S.); palmieri.emilio@hotmail.it (E.P.); giovanni.gambassi@unicatt.it (G.G.); 2 Department of Translational Medicine and Surgery, Catholic University of Sacred Heart, 00168 Rome, Italy; marcello.covino@policlinicogemelli.it; 3 Division of Internal Medicine, Ospedale Santo Spirito in Sassia, 00186 Rome, Italy; maliburzo@gmail.com; 4 Emergency Department, Fondazione Policlinico Universitario A. Gemelli IRCCS, 00168 Rome, Italy; mariella.fuorlo@policlinicogemelli.it; 5 Department of Cardiovascular Sciences, Fondazione Policlinico Universitario A. Gemelli IRCCS, 00168 Rome, Italy; antonio.devita@policlinicogemelli.it

**Keywords:** heart failure (HF), FIB-4 index, heart–liver axis, liver fibrosis, heart failure with preserved ejection fraction (HFpEF), heart failure with mild reduced ejection fraction (HFmrEF), heart failure with reduced ejection fraction (HFrEF)

## Abstract

Liver involvement is closely related to the progression and prognosis of heart failure (HF). This review evaluates the role of FIB-4, a non-invasive and easily calculable index of hepatic fibrosis, as a prognostic biomarker across different HF phenotypes, compared with other established biomarkers of HF. Current evidence demonstrates that elevated FIB-4 scores are closely associated with systemic venous congestion and right ventricular dysfunction and represent a strong independent predictor of adverse cardiovascular events, particularly in HFpEF. In contrast, in HFrEF and HFmrEF, its prognostic value appears less pronounced and more strongly influenced by concomitant primary liver disease, such as MASLD. In acute heart failure, FIB-4 reflects congestion severity and its dynamic reduction during hospitalization carries independent prognostic significance. Compared with NT-proBNP, FIB-4 provides complementary or superior prognostic information in HFpEF, particularly in the presence of predominant right ventricular dysfunction. Additionally, FIB-4 may serve as a screening tool to identify subjects at risk for HFpEF. In conclusion, FIB-4 is a promising tool for risk stratification in HF. However, further prospective studies are needed to establish standardized cut-off values, support its incorporation into clinical guidelines, and clarify its response to SGLT2 inhibitor therapy. Moreover, emerging cardio-metabolic therapies targeting both HF and hepatic fibrosis may further increase the clinical relevance of FIB-4 as a dynamic biomarker of the cardio–hepatic axis.

## 1. Background

Heart failure (HF) is a complex clinical syndrome characterized by high incidence and prevalence rates worldwide. In Western countries, the prevalence of HF is estimated to range between 1% and 3% of the population, and these percentages are expected to increase over time, partly due to population aging [1]. Despite improvements in therapeutic strategies introduced in recent years, patients affected by HF continue to have very high mortality and hospital readmission rates, especially in older age groups, with a considerable impact on healthcare costs [2,3,4]. Therefore, the identification of new risk factors for major cardiovascular events and the development of novel prognostic scores may improve risk stratification and prognosis through earlier diagnosis and more timely therapeutic decisions in high-risk patients.

## 2. Heart–Liver Axis in Heart Failure

**Figure 1:**
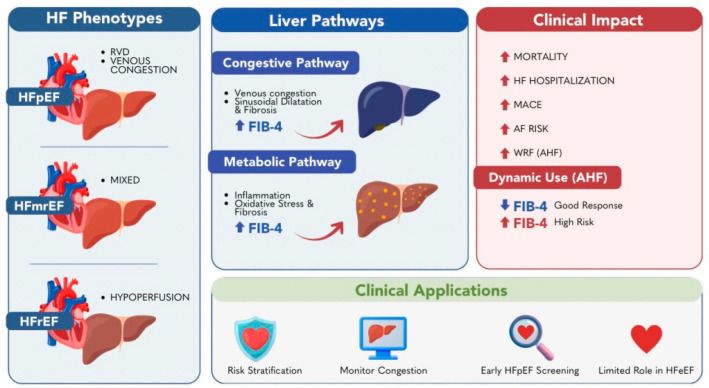
The cardio–hepatic axis in heart failure and the phenotype-dependent prognostic role of FIB-4. In HFpEF, elevated right-sided pressures lead to hepatic congestion and fibrosis, making FIB-4 a strong marker of systemic congestion and prognosis. In HFrEF, liver injury is mainly driven by hypoperfusion, resulting in a weaker and less consistent prognostic value. In HFmrEF, both congestive and ischemic mechanisms contribute to liver injury. In acute heart failure, dynamic changes in FIB-4 reflect the response to decongestive therapy. The coexistence of metabolic liver disease additionally modulates the prognostic value of FIB-4.

The association between HF and liver injury is well established. The pathophysiological mechanism involves right ventricular dysfunction (RVD). Indeed, elevated right atrial pressure is transmitted to the liver through the inferior vena cava and hepatic veins, resulting in congestive liver disease. Right-sided HF leads to hepatic venous congestion with sinusoidal dilatation, hepatic parenchymal oedema and reduced arterial perfusion. The resulting pathophysiological process of centrilobular congestion leads to irreversible structural injury of hepatocytes and progressive fibrosis, which in severe cases may evolve into cardiac cirrhosis (Figure 1) [5,6]. Hepatic involvement is closely related to HF progression and poor prognosis, suggesting that biomarkers of liver fibrosis could also have a prognostic cardiovascular impact [7,8].

However, there are several mechanisms through which liver fibrosis contributes to cardiovascular outcomes, which depend on the underlying etiology of liver damage. In congestive hepatopathy, in particular, prolonged venous congestion results in centrilobular injury and fibrosis, rather than by hepatic inflammation. Persistent elevation of right-sided pressures induces sinusoidal dilatation, presinusoidal edema, and activation of hepatic stellate cells, ultimately leading to fibrosis that develops largely independently of the classical inflammatory cascade [5,6].

In contrast, in patients with concomitant metabolic dysfunction-associated steatotic liver disease (MASLD), hepatic fibrosis develops through a distinct pathway driven by systemic low-grade inflammation, oxidative stress, and endothelial dysfunction. These processes are independently associated with increased cardiovascular risk, accelerated atherosclerosis, and higher rates of cardiovascular morbidity and mortality [9,10].

In clinical practice, these mechanisms may coexist in the same patient with HF, and their relative contribution to hepatic fibrosis may vary across different HF phenotypes.

## 3. Fibrosis-4 Index (FIB-4), Clinical Significance and Rationale in HF

To date, the most reliable method for assessing the degree of liver fibrosis remains liver biopsy. This method represents the gold standard for the assessment of liver fibrosis; however, its use is limited by its invasive nature and the risk of complications such as bleeding or infection [11]. Therefore, several non-invasive methods have been developed to indirectly assess liver fibrosis, including liver stiffness measurement by elastography (Liver Stiffness Measurement—LSM). However, LSM has several limitations, as it is time-consuming and requires dedicated ultrasound equipment [12]. Moreover, measurements may be unreliable in patients with obesity or ascites and may be influenced by concomitant conditions such as hepatic inflammation, portal hypertension, and biliary obstruction [12,13].

Therefore, several non-invasive scores based on serum biomarkers have been developed to overcome these limitations and to estimate both the presence and the severity of liver fibrosis. In 2006 Sterling et al. designed the FIB-4 index to non-invasively estimate the degree of hepatic fibrosis in a cohort of patients with HCV/HIV co-infection [Antiretroviral Therapy in Chronic HIV/HCV Infected Patients Retreatment Trial], with the aim of identifying significant hepatic fibrosis through a simple, non-invasive tool [14]. The developed score is based on a formula incorporating age, platelet count, and the liver enzymes alanine aminotransferase (ALT) and aspartate aminotransferase (AST). The rationale underlying these components reflects the pathophysiology of progressive liver disease: increasing AST levels indicate hepatocellular injury and mitochondrial damage, a decreased platelet count results from portal hypertension, splenic sequestration, and reduced thrombopoietin production [15]. Finally, age reflects cumulative exposure to fibrotic injury. In the validation cohort of Sterling’s original paper [7,14], a cut-off of <1.45 yielded a negative predictive value (NPV) of 90% for excluding advanced fibrosis (Ishak stage 4–6), with a sensitivity of 70%. Furthermore, a cut-off of >3.25 carried a positive predictive value (PPV) of 65% and a specificity of 97%. Using these two thresholds, 87% of the 198 patients with FIB-4 values outside the indeterminate range of 1.45–3.25 were correctly classified, potentially avoiding liver biopsy in 71% of the validation cohort. One year after the index’s introduction, Vallet-Pichard et al. validated the FIB-4 in HCV mono-infected patients, comparing it with liver biopsy and the FibroTest, demonstrating that it served as an inexpensive and accurate marker of fibrosis in HCV infection [16].

When the FIB-4 test began to be used in populations with MASLD, the original cutoff values—derived from a cohort of patients with HIV/HCV coinfection—need to be revised. In fact, MASLD populations tend to be older and have a lower baseline prevalence of fibrosis than viral hepatitis cohorts, rendering the original 1.45/3.25 thresholds less sensitive [17]. Then, in 2019 Castera et al. identified 1.30 as the appropriate lower cut-off for ruling out significant fibrosis (≥F2) in patients with MASLD—a lower threshold than the original 1.45 proposed in the HIV/HCV context [18].

Given these results, the American Association for the Study of Liver Diseases (AASLD) has formally recognized the FIB-4 as the best-validated serological tool for identifying patients with a higher likelihood of advanced liver fibrosis (F3 or F4) in MASLD, recommending it as a first-line screening test given its high negative predictive value. Later, the 2023 AASLD Practice Guidance on MASLD introduced updated cut-off values, specific to the MASLD context: <1.67 for ruling out advanced fibrosis and ≥3.48 for identifying cirrhosis (F4 stage) with high specificity [19].

In recent years, the FIB-4 index, originally validated in hepatological settings, has been increasingly investigated in the context of HF, mainly through observational cohort studies and few RCTs.

Notably, prognostic relevance in HF is indirect and mediated through a multi-step pathophysiological pathway. In fact, HF-related hemodynamic changes, such as elevated right-sided pressures and reduced cardiac output, could lead to hepatic congestion and/or hypoperfusion, which in turn cause hepatocellular injury, fibrogenic remodeling and, ultimately, elevation of the biochemical variables that constitute the FIB-4 formula [15].

Indeed, the presence of advanced liver fibrosis or cirrhosis, expressed as a FIB-4 index > 3.25, has been associated with an increased risk of congestive heart failure (CHF), independently from concomitant infections such as HIV or HCV [20]. Furthermore, elevated FIB-4 values, correlating with a greater degree of venous congestion, have been associated with a significant increase in all-cause mortality in patients with HF [21].

However, its clinical use is often limited by the presence of multiple factors beyond venous congestion that can lead to misleading results.

In fact, the score may be falsely elevated in the presence of systemic inflammatory conditions, acute liver injury, alcohol consumption, or a low platelet count caused by conditions unrelated to the liver. Moreover, one of the most significant structural limitations of the FIB-4 index is the inclusion of age in its formula, which results in a progressive and physiological increase in the score as age advances, regardless of the actual degree of liver fibrosis, often requiring higher diagnostic thresholds for older patients.

Conversely, the index may be falsely reduced in patients who have undergone splenectomy or are on hemodialysis, as these conditions can artificially increase platelet counts or suppress aminotransferase levels.

Because these variables are highly sensitive to non-hepatic physiological changes, clinicians should interpret FIB-4 results with caution and within the clinical context to avoid misdiagnosis [22]. However, as of today, prospective validation and guideline incorporation are still lacking.

This review aims to evaluate the prognostic role of FIB-4 in patients with HF, with particular emphasis on differences across HF subtypes stratified by ejection fractions. Furthermore, FIB-4 will be compared with other biomarkers with an established prognostic role in HF, to assess the potential role of these scores in daily clinical practice.

## 4. Clinical Evidence on the Association Between FIB-4 Index and HF Subtypes

### 4.1. FIB-4 and HF with Preserved Ejection Fraction (HFpEF)

HFpEF currently represents the predominant form of HF, accounting for approximately 50% of all HF cases, and prevalence is projected to increase over the coming years due to population aging [23]. Indeed, it is the most common HF subgroup among elderly patients, women, and individuals with multiple concomitant comorbidities [24]. Moreover, HFpEF is associated with hospitalization and mortality rates that are becoming increasingly comparable to those of heart failure with reduced ejection fraction (HFrEF), probably due to the high burden of comorbidities and frailty in affected patients [25].

**Figure 2:**
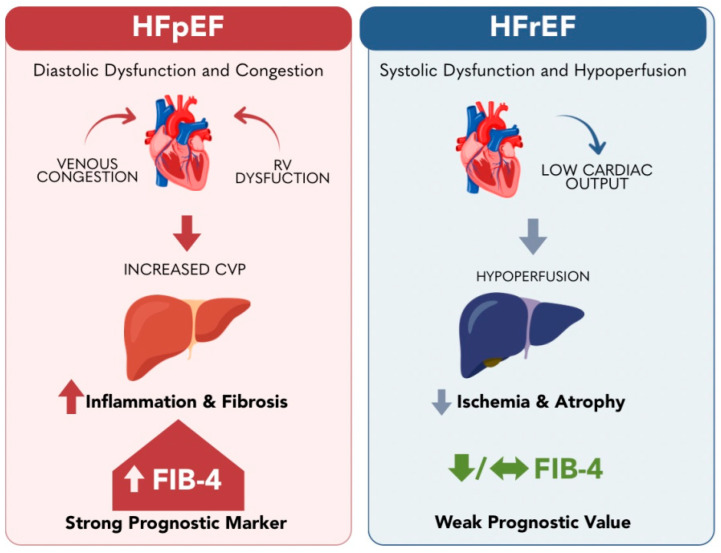
Pathophysiological mechanisms linking FIB-4 to outcomes in heart failure with preserved ejection fraction (HFpEF) and heart failure with reduced ejection fraction (HFrEF). In HFpEF, venous congestion and right ventricular dysfunction lead to increased central venous pressure, promoting hepatic congestion, inflammation, and fibrosis, which result in elevated FIB-4 values that strongly correlate with adverse prognosis. In contrast, in HFrEF, reduced cardiac output and systemic hypoperfusion primarily cause hepatic ischemia and impaired liver function, with a weaker and less consistent association between FIB-4 and clinical outcomes. Overall, FIB-4 appears to be a robust prognostic marker in HFpEF, whereas its predictive value remains more limited in HFrEF.

HFpEF is a complex syndrome characterized by several pathophysiological mechanisms. One of the main hypothesized mechanisms underlying diastolic dysfunction is impaired myocardial relaxation, leading to incomplete relaxation and increased passive myocardial stiffness, resulting in elevated left ventricular filling pressures. These elevated pressures are directly transmitted to the pulmonary circulation, causing both structural and functional changes in the pulmonary arteries, with a progressive increase in both pre-capillary and post-capillary pressures. These pressure changes progressively lead to RVD, which has also been associated with worse prognosis in this group of patients [26,27]. The increase in right-sided pressures in RVD is subsequently transmitted directly to the hepatic venous circulation, eventually leading to hepatic congestion and fibrosis (Figure 2).

In this context, the FIB-4 index may be considered as a prognostic stratification tool in patients with HFpEF, mainly due to its ability to reflect systemic hepatic venous congestion, a condition closely related to cardio-renal dysfunction and HF severity.

The prognostic value of FIB-4 in HFpEF was supported by Takae et al. in a study in which patients hospitalized for AHF were stratified into three subgroups according to FIB-4 index values (cut-off values: <1.3; 1.3–2.67; >2.67). The study demonstrated that, among patients with HFpEF, elevated FIB-4 was an independent predictor of adverse cardiovascular events, with significantly higher risk in the group with FIB-4 > 2.67 compared to the other subgroups. Conversely, this association was not significant in patients with heart failure with mildly reduced ejection fraction (HFmrEF) or HFrEF. Furthermore, the area under the ROC curve (AUC) for predicting major adverse cardiovascular events (MACE) in patients with HFpEF was higher than both that of B-type natriuretic peptide (BNP) and echocardiographically estimated pulmonary artery systolic pressure (PAPs), as well as their respective combinations, suggesting an incremental value of FIB-4 in risk stratification in this patient cohort [28].

Another trial conducted by Nakashima et al. demonstrated an association between elevated FIB-4 values and increased MACE in patients with HFpEF hospitalized for acute heart failure (AHF). In this cohort, FIB-4 values were significantly higher at admission than at discharge, and a reduction in the FIB-4 index during hospitalization was associated with a more favorable prognosis. Accordingly, FIB-4 emerged as an independent predictor of MACE during follow-up, with further increased risk in patients with values > 3.11 before discharge. A significant aspect of the study was that elevated FIB-4 values were correlated with the presence of RVD, assessed using echocardiographic parameters such as tricuspid annular plane systolic excursion (TAPSE) and tricuspid lateral annular systolic velocity (S’), thereby confirming the key role of this pathophysiological component in determining prognosis in patients with HFpEF. Furthermore, N-terminal pro-brain natriuretic peptide (NT-proBNP) was not an independent predictor of MACE, suggesting that FIB-4 provides complementary or superior prognostic information compared with this biomarker in patients with HFpEF and predominant RVD [12].

The FIB-4 also plays an important role in HFpEF, particularly in cases of concomitant fibrosis due to primarily hepatological disease. The important role of liver fibrosis as a prognostic factor in HFpEF was further confirmed by analysis of data from the National Health and Nutrition Examination Survey (NHANES 1999–2018), which evaluated the association between liver fibrosis estimated by non-invasive scores (FIB-4, nonalcoholic fatty liver disease fibrosis score—NFS, AST/ALT ratio) and all-cause mortality. Multivariate Cox regression analysis showed that all considered scores, including FIB-4 with a cut-off value of 1.637, were significantly associated with both total and cardiovascular mortality [29].

A relevant contribution in this regard comes from a study conducted in the TOPCAT trial population, which examined the association between liver fibrosis, particularly in the setting of MASLD, and clinical outcomes in patients with HFpEF. In this cohort, elevated FIB-4 values, indicative of increased risk of significant liver fibrosis, were observed mainly in older and more obese patients and were associated with higher cardiovascular mortality in univariate analysis. Although this association was attenuated in multivariate analysis, FIB-4 nonetheless maintained an independent correlation with the risk of HF hospitalization [30].

One of the factors associated with adverse prognosis in patients with HFpEF is the presence of atrial fibrillation (AF), a comorbidity affecting up to one-third of these patients, particularly the elderly [31,32]. A study conducted by Liu et al. evaluated the association between liver fibrosis, assessed by FIB-4 and NFS scores, and the risk of developing AF in patients with HFpEF. The study demonstrated that patients with advanced fibrosis, defined as FIB-4 > 3.25, had a higher risk of AF, with NFS showing greater predictive value than FIB-4 in multivariate analysis [33]. This suggests that fibrosis scores may indirectly identify additional factors associated with poor prognosis in patients with HFpEF.

Beyond its role in prognostic stratification, FIB-4 may also represent a useful screening tool to identify individuals at risk of developing HFpEF. In this regard, a Japanese study conducted in 710 patients without known cardiovascular disease and with an ejection fraction ≥50% demonstrated that FIB-4 correlated with the HFA-PEFF score, a previously validated diagnostic algorithm for HFpEF, in identifying patients at risk for HFpEF, showing a strong independent correlation between increasing index values and the probability of HFpEF diagnosis [34,35]. Furthermore, FIB-4 was confirmed as an independent predictor of both all-cause mortality and HF hospitalization [35].

The prognostic relevance of FIB-4 in HFpEF may also be explained within the framework of the phenotypic subclassification of this heterogeneous syndrome. According to the classification proposed by Cohen et al., three distinct phenogroups of patients with HFpEF can be identified based on clinical, echocardiographic, and biomarker profiles [36]. Among these, phenogroup 3—referred to as the “cardiometabolic” phenogroup—exhibits the most adverse prognosis and is characterized by a higher prevalence of diabetes mellitus, chronic kidney disease, and obesity, as well as by significant elevation of biomarkers reflecting systemic inflammation (including TNF-α pathway activation) and liver fibrosis in the context of concomitant MASLD. This phenogroup may represent the subgroup in which FIB-4 has the greatest prognostic relevance, owing to the convergence of congestive hepatopathy and metabolic liver disease as dual drivers of hepatic fibrosis. Identifying patients belonging to this phenogroup using a simple, non-invasive index such as FIB-4 may therefore have a direct clinical impact on risk stratification and therapeutic decision-making in HFpEF.

### 4.2. FIB-4 and HFrEF

Among patients with HFrEF the role of the FIB-4 index appears less defined and, in some cases, contradictory to that observed in HFpEF.

Differences between these two HF categories are largely driven by distinct underlying pathophysiological mechanisms, which may also account for the differential prognostic role of FIB-4. Indeed, in HFpEF the primary mechanism underlying liver injury is elevated filling pressures with consequent retrograde hepatic congestion, a feature that has been shown to correlate with FIB-4. In contrast, in HFrEF the contribution of RVD is less pronounced, whereas the predominant pathophysiological mechanism is reduced cardiac output, leading to systemic hypoperfusion and forward hepatic hypoperfusion. This, in turn, may lead to ischemic liver injury, progressing to hepatocellular necrosis and fibrosis [37] (Figure 2).

In a Taiwanese study conducted on patients hospitalized for AHF, the predictive value of FIB-4 for mortality was found to be lower in HFrEF compared with HFpEF or heart failure with mildly reduced ejection fraction (HFmrEF), which are the HF subgroups with a more relevant pathophysiological role of RVD [38].

In the previously mentioned study by Takae et al., unlike patients with HFpEF, the incidence of total cardiovascular events in patients with HFrEF did not differ significantly among groups stratified according to FIB-4 levels (low, intermediate, high), suggesting limited utility of the index in stratifying the risk of MACE in this specific phenotype [28].

However, discordant results emerge from a study by Abdullahi et al., conducted on 4523 patients from the Danish Heart Failure Registry, which showed a significant increase in all-cause mortality among patients with HFrEF who had a moderate or high risk of liver fibrosis, defined according to standard FIB-4 cut-offs [39]. Another trial conducted by Boeckmans et al. demonstrated that FIB-4 was associated with mortality only in patients with HFrEF who had a Fatty Liver Index (FLI) ≥ 60, a parameter indicative of the presence of hepatic steatosis [40]. This aspect differs significantly from HFpEF, in which FIB-4 had a significant predictive role independently of FLI [40].

### 4.3. FIB-4 and HFmrEF

The HFmrEF category is generally regarded as a transitional phenotype with a heterogeneous etiology, sharing features of both HFrEF and HFpEF.

Analysis of baseline characteristics has shown that patients with HFmrEF and HFpEF have, on average, higher FIB-4 values compared with subjects with HFrEF. This observation suggests that the metabolic imprint and the possible component of systemic fibrosis are more strongly represented in the HFmrEF and HFpEF phenotypes than in HFrEF, consistent with a more pronounced inflammatory and metabolic component [28].

Similarly to what has been observed in HFrEF, data regarding the prognostic value of FIB-4 in HFmrEF appear heterogeneous. Some evidence indicates that the FIB-4 index has a more significant predictive value for mortality in patients with HFpEF and HFmrEF compared with those with HFrEF, suggesting a potential role in prognostic stratification also in this intermediate subgroup [38]. Furthermore, this may imply that the pathophysiological mechanisms underlying HFmrEF are more similar to those of HFpEF than to HFrEF, with a greater role of congestive hepatopathy and RVD compared with forward hepatic hypoperfusion.

Conversely, phenotype-based analysis from the trial derived from the Danish Heart Failure Registry showed that in patients with HFmrEF, FIB-4 was predictive of mortality mainly in the presence of concomitant hepatic steatosis, expressed by a FLI ≥ 60 [40]. This finding suggests that, in patients with HFmrEF, the presence of MASLD influences the prognostic value of FIB-4 similarly to what is observed in HFrEF.

## 5. FIB-4 and AHF

The prognostic value of FIB-4 has also been documented in patients hospitalized for AHF, although scientific evidence in this setting remains limited. Acute decompensated heart failure is characterized by a progressive decrease in the effectiveness of natriuretic peptides and persistent activation of neurohormonal pathways, leading to systemic congestion. This hemodynamic condition results in increased central venous pressure and, consequently, increased liver stiffness, providing a plausible pathophysiological rationale for the association between cardiac dysfunction and markers of hepatic fibrosis.

Although available evidence is still limited, Tseng et al. demonstrated that elevated FIB-4 values are associated with worse clinical outcomes even in AHF. In a cohort of 1854 patients hospitalized for AHF, predominantly elderly men, 557 individuals had FIB-4 > 3.25 and this value was associated with increased five-year all-cause mortality. After multivariable adjustment for age, sex, comorbidities, left ventricular ejection fraction, systolic pulmonary artery pressure, left atrial volume, hemoglobin levels, estimated glomerular filtration rate, and serum sodium levels, FIB-4 maintained an independent association with adverse outcomes. For each one standard deviation increase in the score, there was an increased risk of all-cause mortality, rehospitalization for heart failure exacerbation, and cardiovascular mortality [38]. Similarly, a lower FIB-4 threshold (>1.92) was associated with a significant increase in mortality risk [38]. The prognostic value of FIB-4 index persisted even after further optimization of the model including guideline-recommended medical therapy, such as renin–angiotensin–aldosterone system inhibitors, mineralocorticoid receptor antagonists, and beta-blockers [38]. Moreover, a subgroup analysis according to HF phenotype showed that FIB-4 had greater predictive value in patients with HFpEF or HFmrEF, or in the presence of concomitant coronary artery disease (CAD) [38].

Another clinical trial conducted by Tsuda et al. in patients hospitalized for AHF, demonstrated an association between FIB-4 and worsening renal function (WRF), defined as an increase ≥0.3 mg/dL or >25% in creatinine levels from admission to discharge. Indeed, patients with both the highest degree of WRF and FIB-4 values (>2.36) had the highest all-cause mortality rate [41]. Therefore, in patients with AHF, FIB-4 may represent a useful and non-invasive indicator of systemic congestion and fluid overload, which in turn is a major driver of WRF.

Furthermore, it has been shown that in patients hospitalized for AHF, a reduction in FIB-4 values during hospitalization was associated with improved all-cause mortality and reduced risk of rehospitalization within 180 days, suggesting a reduction in systemic venous congestion, similarly to what is observed with BNP [42]. Thus, a combined assessment of trends in FIB-4 and BNP reduction during hospitalization may be useful for prognostic stratification and for evaluating response to diuretic therapy in patients with AHF.

Recent evidence from a prospective cohort study conducted in a Vietnamese population demonstrated that, although the FIB-4 score at admission did not significantly predict in-hospital mortality—possibly owing to the rapid alleviation of hepatic congestion following early intensive medical management—it emerged as a strong independent predictor of 30-day all-cause mortality after discharge. Specifically, after adjustment for relevant clinical variables, each one-point increase in the FIB-4 index was associated with a 9% increase in the risk of 30-day mortality. In the study, patients with FIB-4 scores at or above the threshold of 3.1 had an 82% increased short-term mortality risk. Given that individuals with primary chronic liver disease were excluded, these findings suggest that an elevated FIB-4 primarily reflects secondary hepatic dysfunction driven by the venous congestion and hypoperfusion characteristics of AHF [43].

## 6. FIB-4 vs. Other HF Biomarkers

Among the biomarkers used for the diagnosis and prognostic stratification of patients with HF, natriuretic peptides (NPs) are among the most important. These are a family of peptide hormones produced by ventricular and atrial myocardial cells, as well as by vascular endothelial cells, in response to increased end-diastolic pressure and mechanical wall stress. Among them, NT-proBNP measurement is the most widely used in clinical practice due to its longer half-life and higher circulating concentrations compared with its precursor BNP [44,45].

NT-proBNP has a significant diagnostic value, as levels below 300 pg/mL rule out HF with a negative predictive value of 98% [46]. Moreover, especially in the acute setting, NT-proBNP also has important prognostic value, since elevated levels have been associated with increased mortality and higher rates of HF rehospitalization [45,47,48].

However, NT-proBNP levels may be influenced by various factors, such as advanced age, female sex, presence of AF and chronic kidney disease, which may lead to higher values, or obesity (BMI > 35), in which lower values may be observed [49]. Furthermore, since NT-proBNP mainly reflects volume overload and increased wall stress of the left-sided cardiac chambers, in patients with HFpEF—where the main pathophysiological mechanism is RVD with increased right-sided pressures—NT-proBNP may have a less prominent role and show smaller variations compared with other HF subtypes.

Moreover, as demonstrated in the study by Nakashima et al., NT-proBNP was not an independent predictor of MACE among patients with HFpEF [29]. Therefore, FIB-4 could provide complementary prognostic information compared with NT-proBNP in patients with HFpEF and predominant RVD. Indeed, FIB-4 should be considered a complementary biomarker and not a replacement for NPs such as BNP or NT-proBNP. NPs primarily reflect myocardial wall stress and hemodynamic overload, whereas FIB-4 captures the hepatic consequences of right ventricular dysfunction and systemic venous congestion, as well as the contribution of concomitant metabolic liver disease. Therefore, it may be useful to encourage the combined use of both FIB-4 and NT-proBNP.

Similarly, another recent study conducted on patients with MASLD showed that both elevated FIB-4 values (≥2.67) and NT-proBNP levels (≥125 pg/mL) were associated with increased cardiovascular and all-cause mortality, suggesting the combined use of these markers also for prognostic stratification in clinical practice [50].

Other interesting biomarkers in HF are sST2 and Galectin-3 (Gal-3), which are indicators of myocardial fibrosis and cardiac remodelling. It has been shown that, particularly in HFpEF, Gal-3 levels tend to be elevated and positively correlated with greater diastolic dysfunction as well as increased mortality and hospitalization rates [51,52]. Similarly, sST2 has been shown to be an independent predictor of both mortality and hospitalization in both HFpEF and HFrEF [52,53].

Considering that sST2 and Gal-3 are direct indicators of myocardial fibrosis and remodeling, while FIB-4 reflects the degree of retrograde hepatic congestion secondary to right chamber dysfunction, a combined approach using these biomarker classes could provide complementary and mechanistically distinct information on the cardio–hepatic axis, enabling more comprehensive risk stratification, particularly in patients with HFpEF.

However, to date, direct comparisons between FIB-4 and other fibrosis biomarkers such as sST2 and Gal-3 in heart failure are currently lacking, highlighting a substantial knowledge gap that future prospective studies should aim to address.

Moreover, recent evidence suggested an association between elevated FIB-4 values and the severity of tricuspid regurgitation (TR), which in turn is associated with increased all-cause mortality, further supporting the pathophysiological link between FIB-4 and right-sided cardiac dysfunction. This finding supports the mechanistic link between hepatic venous congestion, right ventricular remodeling, and adverse prognosis that underlies the prognostic relevance of FIB-4 in HF, particularly in phenotypes characterized by predominant RVD.

## 7. Discussion and Clinical Implications

The present narrative review suggests that FIB-4 represents a promising and clinically valuable tool in the management of patients with HF (see Table 1 for a summary of most of the studies supporting this thesis). Given its non-invasiveness, low cost, and ease of calculation, FIB-4 constitutes a practical instrument for prognostic stratification, particularly in the HFpEF subgroup, where the most robust evidence has been reported. Furthermore, owing to its strong correlation with congestion status, FIB-4 may also serve as a useful adjunct for assessing therapeutic response in patients admitted with AHF.

The most robust evidence concerns patients with chronic heart failure and demonstrates differences across the ejection fraction spectrum. Specifically, the correlation between adverse clinical outcomes and elevated FIB-4 values appears stronger in patients with HFpEF than in those with HFmrEF or HFrEF, probably because RVD and retrograde hepatic venous congestion, well reflected by FIB-4, represent the principal underlying pathophysiological mechanisms in this condition. Indeed, in patients with HFpEF, RVD is recognized as an adverse prognostic factor and, correspondingly, elevated FIB-4 values have been associated with a higher risk of mortality and hospitalization for HF.

Furthermore, in patients with HFpEF, FIB-4 may provide complementary and potentially superior information than biomarkers routinely employed in clinical practice, such as NT-proBNP. Indeed, elevated FIB-4 values may identify residual congestion even in the presence of NT-proBNP levels within the normal range. Accordingly, a strategy incorporating the combined use of these non-invasive biomarkers may yield more accurate prognostic stratification in patients with HFpEF. From this perspective, FIB-4 could help to identify patients who may benefit from a more intensive therapeutic approach, including optimization of diuretic therapy, and to enable closer clinical follow-up.

Although, we recommend that future HF cohorts systematically record MASLD status (imaging or FLI) and other confounder liver comorbidities and include them in multivariable models, the prognostic value of FIB-4 in HFpEF is likely greatest in the “cardiometabolic” phenogroup described by Cohen et al. [36]. The pro-inflammatory state associated with liver fibrosis in this subgroup may represent an additional mechanism linking elevated FIB-4 to adverse outcomes [54].

Moreover, one of the pathophysiological mechanisms identified in the development of HFpEF is related to a systemic pro-inflammatory state characterized by elevated circulating inflammatory cytokines (IL-6, TNF-α, sST2—soluble suppression of tumorigenicity-2, pentraxin-3) and increased oxidative stress, which in turn are associated with fibrosis and increased myocardial stiffness [54]. Therefore, the pro-inflammatory state observed in patients with liver fibrosis may be considered an additional factor explaining the link between elevated FIB-4 and adverse prognosis in HFpEF.

In addition to its important prognostic role, FIB-4 also appears to have promising potential as a screening index in the general population to identify patients at risk of developing HFpEF. Indeed, HFpEF remains a diagnostically challenging syndrome, in contrast to HFmrEF and HFrEF, where echocardiographic abnormalities are more readily identifiable. The identification of accessible and reliable tools for early detection in the general population is therefore a research priority.

The association between FIB-4 and clinical outcomes in patients with HFrEF appears less consistent than in other HF phenotypes, although they suggest a potential prognostic role in selected subgroups. Indeed, it can be inferred that the prognostic value of FIB-4 in patients with HFrEF seems to be more strongly influenced by the presence of concomitant MASLD, unlike HFpEF, in which it appears to retain prognostic significance independently of this comorbidity. Different pathophysiologies may account for the different prognostic role of FIB-4 in this subclass compared with HFpEF. As a matter of fact, in HFrEF, anterograde hypoperfusion is largely responsible for pathophysiology, whereas RVD and retrograde hepatic congestion play a subordinate role.

Despite the limited data available, it appears that FIB-4 plays a more important prognostic role in HFmrEF than HFrEF and is more strongly influenced by MASLD than in HFpEF. This finding can be explained mainly by the fact that HFmrEF is an intermediate form of HF between HFpEF and HFrEF from a pathophysiological perspective. However, in this subclass of HF, RVD plays a more significant pathophysiological role than in HFrEF but is certainly less important than in HFpEF.

In conclusion, concomitant liver disease, such as MASLD, seems to have a greater impact on the prognostic role of FIB-4 in patients with HFrEF or HFmrEF. Hence, the FIB-4 may serve as a prognostic indicator for patients with HFrEF only if they have a concomitant history of MASLD, limiting its use in clinical practice as compared to patients with HFpEF.

Although evidence in the acute HF setting is limited, FIB-4 has proven to be a valid indicator, particularly of the degree of systemic congestion. Similarly to BNP, in the acute setting, FIB-4 may be used in clinical practice to assess response to decongestive therapy and to identify higher-risk patients who require more intensive monitoring or closer post-discharge follow-up.

However, no formally validated, HF-specific FIB-4 thresholds exist and no current HF clinical guideline reported FIB-4 as a recommended biomarker for routine risk stratification. In fact, current evidence supports the use of FIB-4 as a prognostic tool in HF, but the optimal cut-offs, timing, and interpretation significantly differ from hepatology practice, and vary by HF phenotype. A 2026 JACC roadmap recommends applying the standard MASLD stepwise algorithm to patients with HF, using FIB-4 thresholds of <1.3 (low risk), 1.3–2.67 (indeterminate risk), and >2.67 (high risk), with the lower threshold increased to <2.0 in patients older than 65 years—but only in stable, non-congested states [55]. This distinction is particularly important, because median FIB-4 values at admission for AHF are substantially higher than those observed in outpatient populations, reflecting acute hepatic congestion and ischemia, rather than established fibrosis [56]. Furthermore, it has been recommended that the FIB-4 score be reassessed after optimized AHF therapy, to determine whether values normalize—suggesting reversible congestion—or remain persistently elevated, indicating possible established fibrosis and the need for hepatology referral [55]. Identification of concomitant primary liver disease may also open additional therapeutic opportunities, as patients with coexisting HF and metabolic dysfunction–associated steatohepatitis (MASH) may benefit from liver-targeted therapies alongside standard HF management.

Indeed, a key area for future research is the potential impact of recently approved therapies for HFpEF on FIB-4 scores and, more broadly, on the cardio–hepatic axis. In fact, several classes of drugs with established or emerging cardiometabolic indications have been shown to affect liver fibrosis. A 2025 meta-analysis of 25 RCTs (*n* = 2600) confirmed that Glucagon-like Peptide-1 Receptor Agonist (GLP-1RAs) significantly reduce liver fat, aminotransferases, and liver stiffness, although class-level histological improvement in fibrosis did not reach statistical significance—evidence of the benefit on fibrosis was stronger for semaglutide and tirzepatide when considered individually [57]. In a network meta-analysis (29 RCTs, *n* = 9324), GLP-1-based polyagonists ranked highest overall for both MASH resolution and fibrosis regression, with pegozafermin (SUCRA 79.9) and survodutide (SUCRA 90.9) among the top-ranked agents [58]. Resmetirom was the first drug approved for non-cirrhotic MASH with F2–F3 fibrosis [59]. Beyond hepatic effects, resmetirom significantly reduced LDL cholesterol, triglycerides, apolipoprotein B, and lipoprotein(a), suggesting potential cardiovascular benefit in a population where cardiovascular disease is the leading cause of death [60]. In addition, fibroblast Growth Factor 21 (FGF21) analogues (pegozafermin, efruxifermin, efimosfermin) have demonstrated particularly strong antifibrotic activity compared to other classes, and a network meta-analysis ranked FGF21 analogues as the most effective class for reducing liver stiffness (SUCRA 82.2) [61]. These agents also improve triglyceride levels, non-HDL cholesterol, and apolipoprotein B concentrations, although direct cardiovascular outcome data remain unavailable [62].

A new drug currently being studied for its cardio-metabolic effects and potential role in reducing liver fibrosis is Lanifibranor, associated with improvement of insulin resistance, glycated haemoglobin (HbA1c), triglycerides, HDL-C, high-sensitivity C-reactive protein (hs-CRP), and blood pressure, with cardiometabolic benefits that appear independent of weight reduction [63]. A phase 3 trial (NATIVE phase 3) evaluating its 72-week efficacy in patients with F2–F3 fibrosis is currently ongoing [64].

Sodium-glucose cotransporter-2 inhibitors (SGLT2i)—namely empagliflozin and dapagliflozin, which have recently received a Class IIa recommendation in the 2023 ESC Guidelines for HFpEF [65]—exert pleiotropic effects that may directly influence the individual components of the FIB-4 formula. Through their natriuretic and decongesting properties, SGLT2i reduce central venous pressure and right-sided filling pressures, potentially attenuating congestive hepatopathy and its downstream fibrogenic stimulus. Moreover, their favourable metabolic effects—including weight reduction, improvement of insulin resistance, and attenuation of hepatic steatosis—may simultaneously reduce the MASLD-related component of hepatic fibrosis. Whether these mechanisms translate into a measurable and clinically meaningful reduction in FIB-4 values, and whether such a reduction is associated with improved cardiovascular outcomes, remains to be established in dedicated prospective studies. Addressing this question would not only strengthen the prognostic role of FIB-4 in HFpEF, but could also establish a non-invasive and readily available biomarker for monitoring the hepatic response to evidence-based HF therapies.

Despite the promising data on the FIB-4 in HF, there are several limitations that warrant careful consideration. Firstly, despite increased interest in recent years, the overall number of available studies remains limited. Secondly, most studies have been conducted in Asian populations, and this limited ethnic and geographical heterogeneity may restrict the generalizability of the findings.

An additional methodological issue concerns an inherent limitation of the FIB-4 formula itself, where age in the numerator results in systematically higher FIB-4 values in older patients, a population known to have both a higher incidence of heart failure and a higher baseline cardiovascular risk. This age-dependent inflation may overestimate the degree of liver fibrosis and inflate the apparent prognostic value of the index in older cohorts. Although some studies have attempted age-specific adjustments or alternative cut-off values for elderly patients, no universally accepted age-corrected threshold has been validated in the HF population to date. This represents an important limitation that should be addressed in future studies.

Additionally, the predominant retrospective design of available studies and the relatively small sample sizes for certain HF subtypes limit the strength of the available evidence. The use of FIB-4 alone does not allow for a definitive distinction between hepatic fibrosis of metabolic origin and congestive hepatopathy secondary to right ventricular dysfunction. Thus, for a more detailed characterization, studies incorporating complementary methods—including liver elastography and, where appropriate, liver biopsy—should be encouraged. Finally, based on current scientific evidence, a definitive and universally validated FIB-4 cut-off value for risk stratification in HF has not yet been established, and the heterogeneity of cut-offs adopted across studies limits direct comparability of results.

## 8. Conclusions and Future Perspectives

FIB-4 has emerging clinical value in the management of patients with HF. As a non-invasive, inexpensive, and readily calculable score, it offers practical utility for prognostic stratification across HF phenotypes, with particular relevance in the HFpEF subgroup. Furthermore, given its robust correlation with congestion status, FIB-4 may serve as an important adjunct for monitoring therapeutic response in patients hospitalized with AHF.

Nevertheless, its prognostic value should always be interpreted within the broader clinical context and integrated with other biomarkers and imaging modalities. To consolidate the role of FIB-4 in HF and support its potential inclusion in clinical guidelines, further studies directly comparing this index with established methods of hepatic fibrosis assessment—both non-invasive, such as liver elastography, and invasive, such as liver biopsy—are warranted. Such evidence would help establish a consensus-based and widely accepted FIB-4 cut-off value in the HF setting.

Whether SGLT2 inhibitors—through their decongestant and metabolic effects on the cardio–hepatic axis—may produce a clinically meaningful reduction in FIB-4 values and whether such a reduction carries prognostic significance, remain open questions that dedicated prospective studies should address. Future prospective studies should also clarify whether emerging HF and cardio-metabolic therapies may modulate FIB-4 values and whether these changes translate into improved cardiovascular outcomes.

## Figures and Tables

**Table 1:** Summary of studies evaluating the prognostic role of FIB-4 in heart failure.

Author (Year)	Study Design	Population (Setting, Country/Region)	HF Phenotype	FIB-4 Cut-off	Primary Outcome	Main Results (FIB-4)
Nakashima et al., 2021 [12]	Observational	AHF and pEF; Japan	HFpEF	>3.11	MACE	Independent predictor: reduction during hospitalization associated with improved outcomes; correlation with RVD
So-Armah et al., 2017 [20]	Cohort	Veterans with HF and HIV+/−; USA	Incident HF	>3.25	HF incidence	Advanced fibrosis associated with incident HF
Takae et al., 2021 [28]	Observational	AHF; Japan	HFpEF vs. HFrEF/HFmrEF	<1.3; 1.3–2.67; >2.67	MACE	Independent predictor only in HFpEF, superior to BNP and PAPs
Guo et al., 2024 (NHANES) [29]	Population-based cohort	HF; USA, multi-ethnic	Not stratified by EF	1.637	All-cause and CV mortality	Independently associated with both CV and all-cause mortality
Peters et al., 2021 (TOPCAT) [30]	Post hoc RCT analysis	HFpEF; USA and Europe	HFpEF	Continuous	HF hospitalization CV mortality	Independently associated with HF hospitalization
Liu et al., 2022 [33]	Observational	HFpEF	HFpEF	>3.25	Incident AF	Not associated
Okamoto et al., 2023 [35]	Observational	HFpEF in general population; Japan	HFpEF screening	Continuous	HFpEF diagnosis, mortality	Correlates with HFA-PEFF score; independent predictor of mortality
Tseng et al., 2022 [38]	Cohort	AHF; Asia	All (stronger in HFpEF/HFmrEF)	>3.25; >1.92	Mortality Rehospitalization	Independent predictor of adverse outcomes, stronger in HFpEF/HFmrEF
Mohamed et al., 2025 [39]	Registry-based cohort	HF hospitalization; Denmark	HFrEF	Conventional (1.45/3.25)	All-cause mortality	Moderate/High FIB-4 associated with increased mortality
Boeckmans et al., 2025 [40]	Prospective cohort	HF ± MASLD; Europe	HFrEF/HFmrEF	Conventional (1.45/3.25)	Mortality	Predictive only in association with steatosis (FLI ≥ 60)
Tsuda et al., 2023 [41]	Observational	AHF	All	>2.36	WRF Mortality	Higher FIB-4 associated with WRF and increased mortality
Truyen et al., 2026 [43]	Cohort	AHF	All	>3.1	30-day all-cause mortality	Each one-point increase in FIB-4 raised the 30-day mortality risk by 9%

Abbreviations: HF, heart failure; AHF, acute heart failure; HFpEF, heart failure with preserved ejection fraction; HFrEF, heart failure with reduced ejection fraction; HFmrEF, heart failure with mildly reduced ejection fraction; EF, ejection fraction; FIB-4, Fibrosis-4 Index; BNP, B-type natriuretic peptide; PAPs, Systolic Pulmonary Arterial Pressure; FLI, Fatty Liver Index; HFA-PEFF score, Heart Failure Association Pre-test assessment, Echocardiography & natriuretic peptide score; MACE, major adverse cardiovascular events; AF, atrial fibrillation; WRF, worsening renal function; CV, cardiovascular; RCT, randomized controlled trial; NHANES, National Health and Nutrition Examination Survey; HIV, human immunodeficiency virus; MASLD, metabolic dysfunction-associated steatotic liver disease.

## Data Availability

No new data were created or analyzed in this study.

## Abbreviations

The following abbreviations are used in this manuscript:

AF	Atrial Fibrillation
AHF	Acute Heart Failure
ALT	Alanine Aminotransferase
APRI	Aspartate Aminotransferase to Platelet Ratio Index
AST	Aspartate Aminotransferase
HbA1c	Glycated Hemoglobin
Hs-CRP	High-sensitivity C-Reactive Protein
AUC	Area Under the Curve
BMI	Body Mass Index
BNP	B-type Natriuretic Peptide
CAD	Coronary Artery Disease
CHF	Congestive Heart Failure
CV	Cardiovascular
EF	Ejection Fraction
ESC	European Society of Cardiology
FGF21	Fibroblast Growth Factor 21
FLI	Fatty Liver Index
FIB-4	Fibrosis-4 Index
Gal-3	Galectin-3
GLP-1RAs	Glucagon-like Peptide-1 Receptor Agonist
HF	Heart Failure
HFmrEF	Heart Failure with Mildly Reduced Ejection Fraction
HFpEF	Heart Failure with Preserved Ejection Fraction
HFrEF	Heart Failure with Reduced Ejection Fraction
HFA-PEFF	Heart Failure Association Pre-test Assessment, Echocardiography & Natriuretic Peptide Score
HIV	Human Immunodeficiency Virus
HCV	Hepatitis C Virus
IL-6	Interleukin-6
LSM	Liver Stiffness Measurement
MACE	Major Adverse Cardiovascular Events
MASLD	Metabolic Dysfunction-Associated Steatotic Liver Disease
MASH	Metabolic Dysfunction–Associated SteatoHepatitis
MAFLD	Metabolic-Associated Fatty Liver Disease
NFS	Nonalcoholic Fatty Liver Disease Fibrosis Score
NHANES	National Health and Nutrition Examination Survey
NPs	Natriuretic Peptides
NPV	Negative Predictive Value
NT-proBNP	N-terminal pro-B-type Natriuretic Peptide
PAPs	Pulmonary Artery Systolic Pressure
PPV	Positive Predictive Value
RCT	Randomized Controlled Trial
ROC	Receiver Operating Characteristic
RVD	Right Ventricular Dysfunction
SGLT2i	Sodium-Glucose Cotransporter-2 Inhibitors
sST2	Soluble Suppression of Tumorigenicity-2
SUCRA	Surface Under the Cumulative Ranking Curve
TAPSE	Tricuspid Annular Plane Systolic Excursion
TNF-α	Tumor Necrosis Factor Alpha
TR	Tricuspid Regurgitation
WRF	Worsening Renal Function
